# Evaluating the Impact of Environmental Exposure on the Performance of Polyester Sportswear Materials

**DOI:** 10.3390/polym17121616

**Published:** 2025-06-10

**Authors:** Ivana Salopek Čubrić, Antonija Petrov, Goran Čubrić

**Affiliations:** 1 Department of Textile Design and Management, Faculty of Textile Technology, University of Zagreb, Prilaz baruna Filipovića 28 a, 10000 Zagreb, Croatia; ivana.salopek@ttf.unizg.hr; 2 Department of Clothing Technology, Faculty of Textile Technology, University of Zagreb, Prilaz baruna Filipovića 28 a, 10000 Zagreb, Croatia; antonija.petrov@ttf.unizg.hr

**Keywords:** polyester, yarn, sportswear, comfort, aging, knitted fabric, bending, elongation, abrasion, wetting, drying

## Abstract

The growing popularity of sporting activities has led to an increased demand for sportswear. Consequently, sportswear developers are, therefore, faced with the challenge of meeting the increasing and more demanding expectations of users. In this study, the effects of aging on sportswear materials made from recycled and conventional polyesters are investigated. The properties analyzed include mass per unit area, thickness, porosity, elongation, bending stiffness, abrasion, and wetting and drying behavior. The results showed that aging leads to an increase in fabric thickness, with recycled polyester showing the largest increase of 6.6%, as well as a reduction in porosity. In addition, recycled polyester exhibited the highest stiffness, while conventional polyester, with the lowest mass per unit area, had the lowest stiffness and offered greater flexibility in movement. Non-aged samples had a shorter wetting time, while the aged materials dried faster. As the material ages, its abrasion resistance decreases. However, the recycled polyester material showed better wear resistance after aging compared to standard materials, indicating its potential long-term durability. In summary, the results suggest that aging significantly affects the structure and functional properties of fabrics, which is important for designing durable sportswear that maintains optimal performance over time and use.

## 1. Introduction

Sport plays a significant role in societies around the world, not only as a form of recreation and entertainment but also as a driver of health, social integration, and economic activity. With its immense global popularity, particularly in disciplines such as football, the demands on sportswear have increased, necessitating the development of high-performance materials tailored to the physiological and biomechanical needs of athletes. Consequently, sportswear developers are faced with the challenge of meeting the growing and ever-increasing expectations of users [1,2]. Sportswear fabrics are usually made of polyester or polyamide in combination with elastane [3,4]. In addition, the use of recycled materials has gained importance in terms of environmental sustainability, leading to continuous research into innovative blends and eco-friendly alternatives to improve both performance and sustainability [5,6].

Despite its relevance, the design of sportswear materials has not yet received adequate scientific attention, opening a promising area for future research. Several studies emphasize that wearer comfort plays an important role in the design and development of sportswear [7,8,9,10]. During intense physical activity, the human body produces a considerable amount of sweat, which must be effectively wicked away to ensure an optimal level of comfort.

One of the key factors influencing the comfort and functionality of sportswear is its ability to dry quickly. This feature keeps the wearer dry and comfortable during intense sport activity. As a result, some researchers have studied the properties and types of materials that significantly impact drying performance [11,12]. Quick-drying fabrics are particularly important for athletes, as they help to regulate body temperature and minimize the risk of skin irritation and infections. These fabrics often incorporate advanced textile technologies that enhance moisture wicking and evaporation. Additionally, some textiles undergo special treatments or coatings to further enhance their moisture management capabilities. Recent studies have explored various innovations in polyester fabric technology aimed at optimizing drying efficiency. For instance, one study compared the drying performance and environmental impact of biodegradable polyester yarn with conventional yarn. This showed that biodegradable variants not only dry more efficiently but also improve the comfort of the clothing, especially after repeated washes [13]. A group of authors reviewed existing research on how fabric properties, physiological factors, and environmental conditions impact the drying performance of sportswear. Their analysis identified key knowledge gaps and suggested enhancing future research interest in order to increase fabric performance [14]. The porosity of a material is also an important property, as it may significantly affect its drying process by allowing moisture to pass through the structure more quickly, thereby enhancing drying efficiency. This property is particularly important for active sportswear, as efficient moisture transport improves comfort and performance. However, increased porosity also leads to higher sweat permeability, which can influence the garment’s ability to maintain optimal comfort during physical activity. Scientific research has extensively examined the link between fabric porosity and drying performance, emphasizing its dual role in moisture management and wearer comfort [15,16].

Athletes frequently encounter external factors, like high temperatures, solar radiation, precipitation, and wind, all of which influence the properties of sportswear materials. Continuous use leads to wear and tear, sweat absorption, and material degradation from regular maintenance, negatively affecting textile performance and durability and altering its physical and mechanical properties. Previous research [1,17] has shown that degradation processes in various materials can lead to reduced elasticity, changes in thickness and mass per unit area, and alterations in liquid absorption and drying properties. Several studies have examined the impact of aging on polymer performance [6,18,19,20,21]. The findings of one of these studies [6] indicated that although the surface properties of the material remained unchanged by aging, the tensile strength decreased by up to 26% for conventional polyester and 15% for recycled polyester. Recycled polyester materials also exhibited a greater loss of mass due to abrasion, a 23% reduction in moisture absorption, and a 30% increase in wetting time. A follow-up study [21] also found undesirable changes in material properties due to aging, highlighting the need for a deeper understanding of polymer behavior in certain environments.

In summary, the ideal materials for sportswear are those that quickly absorb moisture, dry rapidly, do not significantly change other properties, and possess antibacterial properties to prevent unpleasant odors and potential infections. Therefore, sportswear design must consider these factors to ensure optimal functionality and comfort for the user during physical activities [22,23,24,25]. While advancements have been made in the development of high-performance sportswear, research on the aging of these materials is still quite limited. Key gaps include the effects of aging on material performance, property retention (primarily physical–mechanical properties), and sustainability issues (related to the use of recycled materials and their effectiveness). Addressing these gaps could drive innovation in materials science and improve both the durability and environmental sustainability of future sportswear products. Moreover, there is a notable lack of targeted research focusing on football-specific garment performance, especially regarding the impact of material aging due to prolonged use, washing, and exposure to external factors that specifically characterize this sport. This gap limits the ability to ensure long-term comfort, durability, and performance. Therefore, further research is essential not only to improve the functional design of football sportswear but also to provide meaningful feedback to manufacturers, elite athletes, and the scientific community. Such efforts can stimulate innovation, inform evidence-based product development, and inspire broader scientific engagement in the field of sport-specific materials research.

The aging of sportswear materials requires a specific approach, as they undergo different types of degradation compared to general polymeric materials. In our previous research, the aspect of sportswear material degradation was analyzed [21]. The results of the study indicated that the degradation of the investigated materials did not occur over a simulated period of use. Therefore, the investigation in this paper focuses on the changes in the physical–mechanical properties of materials used for football sportswear due to material aging, focusing on eight important material properties. More specifically, this study investigates how aging affects various properties of both recycled and conventional polyester materials, such as mass per unit area, thickness, porosity, elongation, bending stiffness, wetting, drying, abrasion, and piling. In contrast to aging tests of polymers in the laboratory, which are carried out under controlled conditions, this study replicates real-life usage scenarios and analyses how aging affects key performance characteristics. The aim is to gain a comprehensive understanding of how material performance evolves over time to support the development and selection of sportswear materials for the world’s most popular sport. These insights will improve durability and functional properties to help athletes perform better.

## 2. Materials and Methods

### 2.1. Material Selection

For the purpose of this study, a set of four different knitted fabrics commonly used in the production of football jerseys was selected. These materials were sourced from renowned and reputable manufacturers to ensure that all specimens were representative of high-quality, commercially available products in the sportswear market. The focus was placed on synthetic polymers most commonly used in athletic apparel in order to obtain results that were as relevant and applicable to real-life conditions as possible. This selection strategy aimed to approximate actual usage scenarios and allow for an accurate assessment of the materials as they are worn during sports activities.

Among the four fabrics selected, one fabric was made from recycled polyester. Its inclusion in the study was motivated by the increasing importance of sustainability in the textile and sportswear industry, as well as the need to compare its behavior with that of conventional polyester fabrics under controlled test conditions. Although the availability of recycled alternatives was limited during the procurement phase, the selected recycled sample was the only one accessible that met the study’s structural and performance criteria. This allowed for a preliminary assessment of the differences in structural stability and aging response between recycled and standard polyester fabrics, providing valuable insight into the suitability of recycled materials for use in high-performance sportswear. The recycled material used in the investigation was derived from post-consumer articles, which are collected, cleaned, shredded, and processed into fibers through mechanical recycling.

Details of the selected materials are provided in Table 1, including their mass per unit area, thickness, horizontal and vertical stitch density, and knitted structure.

### 2.2. Material Aging Protocol

The selected materials were subjected to an aging protocol specifically designed to simulate the realistic conditions that professional footballers are exposed to during both training and official matches. The aim of this protocol was not to isolate individual environmental factors, such as atmospheric exposure or washing, but rather to replicate a comprehensive set of real-life conditions that sportswear is commonly exposed to in practice. By applying this integrative approach, the study provides a more representative evaluation of the overall material behavior under actual conditions of use.

The aging procedure was carried out over a four-week period (20 days, totaling 80 h) during the summer season in a temperate continental climate (45°48′055.4364″ N, 15°57′059.6448″ E), with direct environmental exposure. The atmospheric conditions during this aging period were continuously monitored and are summarized in Table 2. These measured conditions were found to be consistent with the general forecasts provided by the European Meteorological Centre’s weather forecast model HRES [26]. To realistically mimic usage patterns of football apparel, the aging protocol was divided into two daily exposure sessions—morning and mid-morning—reflecting common timeframes of training and match activity.

The morning session took place from 8:00 a.m. to 10:00 a.m., with the materials exposed to direct sunlight for two hours. After 15 min of exposure, an artificial sweat solution was applied to the materials to simulate the onset of perspiration during physical exertion. The solution was prepared using acidic sweat powder (pH 5.5), in accordance with ISO 105-E04 [27]. It was applied evenly across both sides of the fabrics in ten successive spraying cycles to ensure uniform distribution and simulate progressive perspiration during use.

The mid-morning session followed immediately from 10:00 a.m. to 12:00 p.m. Similar to the first phase, the materials were again exposed to sunlight, and a second application of artificial sweat was administered 10 min into the session. This phase was intended to simulate conditions typical of a football match, where intensive physical activity leads to continuous sweat production and mechanical stress on the garment.

In addition to solar and sweat exposure, daily washing was included as an essential part of the protocol to simulate regular garment maintenance. Each material was washed at the end of every day in a standard household washing machine using a phosphate-free and optical-brightener-free detergent. The washing cycle was set at 30 °C, a temperature selected to avoid thermal degradation of the textile structure while ensuring effective removal of sweat residues and dirt. After washing, all materials were dried in a controlled indoor environment, protected from direct sunlight and external environmental factors, to prevent any additional degradation caused by UV exposure or weather conditions. This aspect of the protocol was included to reflect real-world laundering practices and to account for the cumulative effects of daily care on the performance of the materials. By combining sun exposure, sweat simulation, and repeated washing, the protocol aimed to closely approximate the actual conditions encountered by sportswear in real-life scenarios, providing a realistic framework for evaluating aging-induced changes in fabric properties. While this study does not differentiate between the effects of individual aging factors (e.g., atmospheric conditions vs. washing), the chosen approach enhances ecological validity and ensures that the findings are applicable to practical conditions of use.

### 2.3. Measurement Methods

In the experimental part of this paper, the following properties of the knitted fabrics were tested: mass per unit area, thickness, porosity, wetting and drying behavior, bending stiffness, abrasion, and elongation. These tests were carried out to determine the changes caused by the aging process of the material. Each test method and the results obtained are described in detail below.

#### 2.3.1. Mass per Unit Area and Thickness Testing

Mass per unit area is a key parameter in the characterization and comparison of knitted fabrics. The measurement procedure was conducted according to the HRN ISO 3801:2003 [28] standard. Samples measuring 100 ± 2 × 100 ± 2 mm were cut and weighed using an analytical balance (Kern ALJ 220-4, Kern & Sohn GmbH, Balingen, Germany) with a precision of 0.0001 g. Based on the measured values, the mass per unit area was calculated and expressed in grams per square meter (g/m^2^).

The method described in ISO 5084 [29] was used to determine the thickness of the material. A thickness gauge was used for this purpose, namely, the DM-2000 model (Wolf Messtechnik GmbH, Freibergu, Germany). Before the test, the specimens were conditioned according to ISO 139 [30] to ensure standard atmospheric conditions. During the test, each specimen was subjected to a pressure of 1 kPa over an area of 20 cm^2^. For each measurement, the thickness gauge was placed vertically between the reference plate and the parallel circular plate covering the sample. The thickness measurement test areas were strategically placed in the center of the 20 cm^2^ sample, avoiding the edges. These test areas are arranged diagonally, starting from the lower left corner of the sample. A total of 10 measurements were taken at different locations on the specimen to obtain accurate readings and the average value was calculated.

#### 2.3.2. Porosity Testing

The porosity of the knitted fabric was determined from microscopic images of the specimen taken with a Dino-Lite Pro Hr AM7000/AD7000 series digital microscope (Dino-Lite, Almere, The Netherlands). For each specimen, a total of 10 images were taken at different locations on the fabric. To determine the porosity, DinoCapture 2.0 software was used. The software allows a very accurate determination of the surfaces of the voids within the loops in relation to the total area of the fabric. The porosity was calculated from the measured void surfaces.

#### 2.3.3. Wetting and Drying Testing

An infrared thermographic camera, the TESTO 872 (Testo Inc., Titisee-Neustadt, Germany), was used to determine the wetting and drying time of the materials. This device is equipped with practical functions that enable the generation of objectively comparable infrared images with minimal errors. The TESTO ε-Assist and TESTO ScaleAssist features were used to avoid measurement uncertainties and to easily achieve optimal emissivity (ε) and reflected temperature (RTC) settings for thermography and thermal imaging scales. The experiment took place in a controlled environment at a temperature of 20 ± 2 °C and a relative humidity of 65 ± 3%. Round metal frames were used to secure the samples, with a skin-simulating foil first positioned within the frames, followed by the fabric samples. The frames were secured to prevent any movement of the samples.

A micropipette was used to apply 0.1 mL of the prepared artificial sweat solution (acidic sweat powder with a pH of 5.5, prepared according to ISO 105-E04 [26]) onto each sample. The wetting time, defined as the time required for the liquid to be absorbed by the material, was recorded. Afterward, the samples were enclosed within a box that had an opening at the top, allowing the infrared thermographic camera to continuously monitor the drying process.

The infrared thermographic camera captured images throughout the entire drying period, and thermograms were recorded every 10 min to clearly observe the changes. Two samples were placed inside the box: the non-aged sample on the left and the aged sample on the right to immediately observe the effects of aging.

After the test was completed, the obtained thermograms were analyzed using the online program Photo Measure to calculate the wetting surface of each sample. As an additional verification, the wetting surface was calculated as follows:

(1) $$A=\frac{Wd_{x}\;\cdot \;Wd_{y}\;\cdot \;\pi }{4}$$

where *A* is the wetting surface (mm^2^), *Wd_x_* is the wetting diameter in the direction of the *x*-axis (mm), and *Wd_y_* is the wetting diameter in the direction of the *y*-axis (mm).

Further calculations provided the wetting speed according to the following formula:

(2) $$Ws_{x}=\frac{Wd_{x}}{Wt}$$

(3) $$Ws_{y}=\frac{Wd_{y}}{Wt}$$

where *Ws_x_* is the wetting speed in the direction of the *x*-axis (mm/s), *Ws_y_* is the wetting speed in the direction of the *y*-axis (mm/s), *Wd_x_* is the wetting diameter in the direction of the *x*-axis (mm), *Wd_y_* is the wetting diameter in the direction of the *y*-axis (mm), and *Wt* is the wetting time (s). Finally, the total time required for the sample to completely dry was recorded.

#### 2.3.4. Bending Stiffness Testing

The cantilever method for measuring fabric stiffness is a widely used technique to assess the rigidity and flexibility of a fabric. This method is straightforward, providing a quantitative measure of the fabric’s resistance to bending. The method itself uses the engineering principles of beam theory, and the fabric is made to deform under its own weight as a cantilever. Within this method, the focus is on the measurement of the fabric bending length that is defined through the interaction between the fabric bending stiffness and the fabric mass per unit area [31], i.e.,

(4) $$c=l{\left(\frac{cos\frac{\theta }{2}}{8tan\theta }\right)}^{\frac{1}{3}}\approx \frac{1}{2}$$

where *c* is bending length and l is cantilever length.

For the measurement, the rectangular samples of 50 × 150 mm were cut in both wale and course directions from each fabric investigated. The specimen was placed on a plane horizontal platform and moved forward to project as a cantilever from it. The cantilever length of the fabric was measured at the moment when the straight line connecting the edge of the plane horizontal platform and the edge of the specimen made an angle of 41.5°. The bending stiffness was further calculated as follows:

(5) $$B=m\cdot c^{3}$$

where *B* is bending stiffness, *m* is the mass per unit area of the fabric, and *c* is bending length.

#### 2.3.5. Abrasion and Piling Testing

To further evaluate the performance and durability of the selected materials under conditions simulating real-life wear, both abrasion and pilling resistance tests were conducted on samples before and after the aging process. The abrasion tests were carried out using the AquAbrasion—Wet and Dry Abrasion Tester (James Heal, Halifax, UK), an advanced version of the Martindale device equipped with a precision liquid delivery system.

The abrasion resistance was assessed in accordance with the ISO 12947-3:2008 standard [32]. The test specimens (Pr, Ps1, Ps2, Ps3) had a round shape with a diameter of 38 ± 5 mm and were mounted on holders to undergo abrasion against a standard woven woolen fabric (abrasive material) positioned on a felt base with a diameter of 140 ± 5 mm. A normal pressure of 9 kPa was applied, and the movement followed a Lissajous pattern, enabling the holder to rotate freely around its vertical axis.

In line with the requirements of high-performance sportswear, where materials are expected to endure frequent and intense use, mass loss was chosen as the key parameter to evaluate abrasion resistance. The specimens were weighed before and after completing 5000 abrasion cycles, and the mass change was recorded to quantify the abrasion.

The tendency of knitted fabrics to form surface pilling was evaluated on both aged and non-aged samples in accordance with ISO 12945-2:2020 [33]. Visual assessment was conducted by comparing the surface appearance of the specimens after abrasion to that of the original, unabraded samples, following the grading procedure described in ISO 12945-4:2020 [34]. The grading scale ranges from grade 1 to 5, with grade 5 indicating no visible pilling and grade 1 indicating severe surface pilling.

This combined approach allowed for a comprehensive assessment of material degradation in response to mechanical stress, enabling a better understanding of the long-term behavior and suitability of the tested fabrics for use in high-intensity sports applications, such as football jerseys.

#### 2.3.6. Elongation Testing

The elongation properties of knitted fabrics were measured using the Statimat M tensile tester (Textechno, Mönchengladbach, Germany) at a constant elongation rate in accordance with ISO standard 13934-1:2013 [35]. Rectangular samples 50 mm wide and 200 mm long were prepared for the tests. The experiment was conducted in a controlled environment with a temperature of 20 ± 2 °C and a relative humidity of 65 ± 3%. A total of five measurements were taken in the wave and course direction, and the mean value was used.

## 3. Results and Discussion

### 3.1. Results of Mass per Unit Area and Thickness

Table 3 presents the mass per unit area of the tested knitted materials before and after the applied aging protocol, along with the corresponding absolute and relative changes. All tested materials exhibited an increase in mass per unit area after aging, with the most prominent change observed in sample Ps2 (8.33%), followed by Ps1 (5.67%), Pr (2.26%), and Ps3 (0.68%). This increase can be attributed to the combined effects of repeated exposure to artificial sweat, daily washing, and structural changes within the fabric. Although polyester is a hydrophobic fiber, residues from sweat and detergent may become partially trapped in the fabric structure over time. In addition, the mechanical stress caused by repeated washing and exposure to environmental factors can induce fiber relaxation and loop compaction, resulting in a denser arrangement of the textile structure. The extent of mass increase also appears to be influenced by the knit structure, as more compact or interlocked constructions may retain more residues and exhibit greater structural tightening, contributing to higher values of mass per unit area after aging.

Table 4 shows the values of material thickness before and after aging for selected materials. An increase in thickness can be seen for all materials after aging. The thickness of the material Pr, made from 100% recycled polyester, increased by approximately 6.6%, while the absolute change amounted to +0.030 mm. The material Ps1 shows an increase of approx. 4.2% (+0.026 mm); Ps2 increased by approx. 4.3% (+0.021 mm) and Ps3 by 5.6% (+0.031 mm). The observed increase in thickness after aging can be attributed to various structural changes. As materials age, there is often a relaxation and compression of the fibers, which contributes to an overall increase in material volume. Furthermore, the results are consistent with the behavior of the materials discussed in the previous work [6] and confirm that aging has a significant impact on the shrinkage of the material. This increase in thickness can also be attributed to a greater number of protruding fibers on the surface of the fabric. In addition, both chemical and mechanical recycling processes can have a negative impact on the physical and chemical properties of recycled materials. These processes may lead to structural changes that increase the susceptibility of the fabric to moisture interaction and aging effects [36]. The combination of these factors emphasizes the complexity of thickness changes in materials exposed to aging and recycling.

A statistical analysis was performed using a *t*-test to determine whether there was a statistically significant difference in thickness change between the recycled polyester material (Pr) and the conventional materials (Ps1, Ps2, and Ps3) after aging. The results showed that there was no statistically significant difference in thickness change between Pr and Ps2 (*p* = 0.0556), as the *p*-value exceeded the significance threshold (α = 0.05). However, a statistically significant difference was observed between Pr and both Ps1 (*p* = 0.0227) and Ps3 (*p* = 0.0052), indicating that aging had a notable effect on the thickness changes of these materials. These findings suggest that differences in material properties may contribute to the variations in thickness change after aging.

### 3.2. Results of Material Porosity Testing

**Figure 1:**
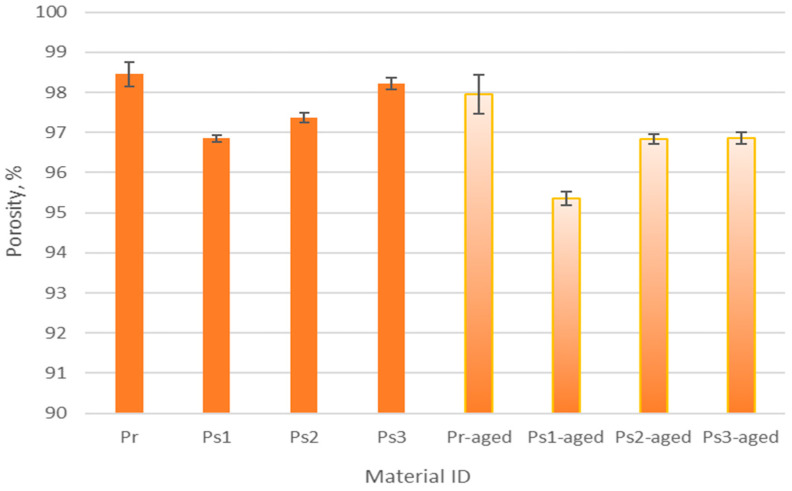
The porosity of tested materials.

Figure 1 presents the porosity of the tested materials before and after aging. As seen, the porosity of the non-aged materials ranged from 96.85% to 98.45%, while that of the aged materials ranged from 95.36% to 97.95%. The graph indicates that aging reduced the porosity of both recycled and conventional polyester fabrics. Notably, the recycled polyester material (Pr) initially exhibited the highest porosity at 98.45%, which decreased to 98.06% after aging, whereas the conventional polyester material (Ps1) started with the lowest porosity at 96.85%, decreasing to 95.36% after aging. The primary cause of the reduction in porosity after aging is to be explained by material shrinkage. This shrinkage was verified by an increase in material thickness, as analyzed in the previous chapter. This finding aligns with the results of a prior study [37]. Both aged and non-aged materials composed of recycled yarn exhibited a higher standard deviation in porosity values. This increased deviation signifies greater inconsistency and variability in the recycled polyester material, which could impact its performance and overall quality in its intended application.

### 3.3. Results of Wetting and Drying

**Figure 2:**
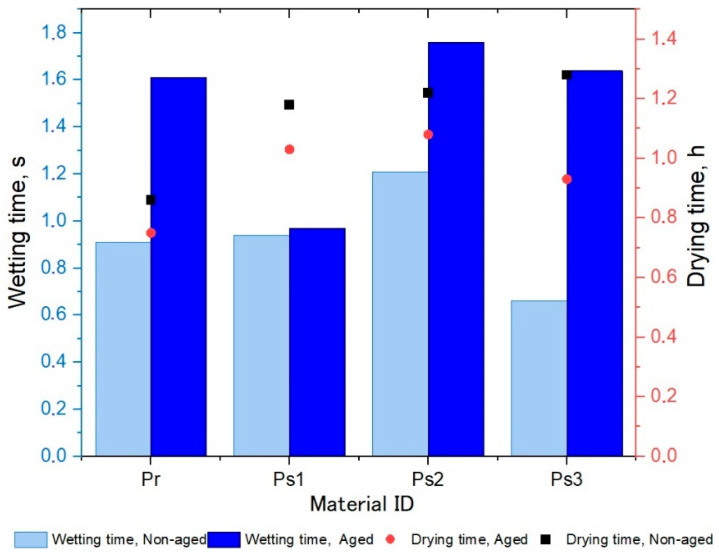
Comparison of wetting and drying times for non-aged and aged materials.

Figure 2 provides a comparative analysis of the wetting and drying times for selected specimens. The wetting time, shown in seconds on the left *y*-axis, increased for all materials when they were aged (dark blue bars) compared to their non-aged counterparts (light blue bars). Among them, material Ps2 showed the highest wetting time both in the aged and non-aged states. The drying time, indicated by circular and square markings on the right *y*-axis in hours, remained relatively consistent for the non-aged specimens Ps1, Ps2, and Ps3, while Pr exhibited the shortest drying time (black squares). After aging, the drying time decreased noticeably for all specimens, with the most significant decrease observed for sample Ps3, where the drying time decreased by 27.34% (red circles). This indicates that aging significantly affects the wetting and drying behavior of the textiles and leads to changes in the material properties.

**Figure 3:**
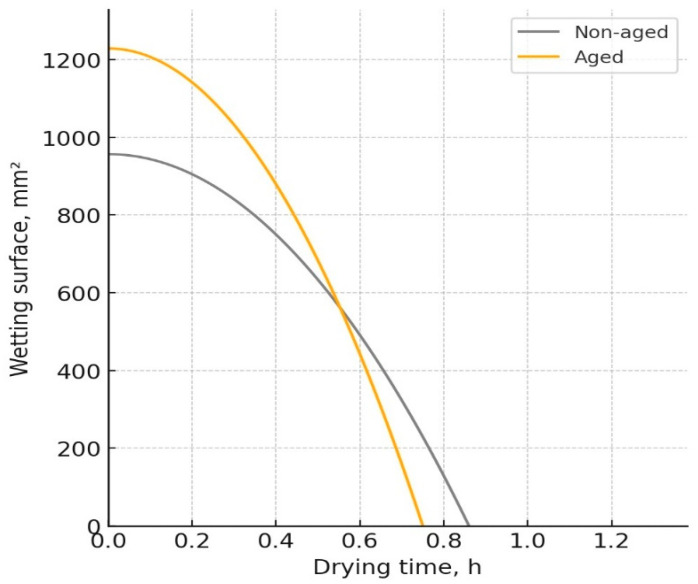
W-D profile of the fabric Pr.

**Figure 4:**
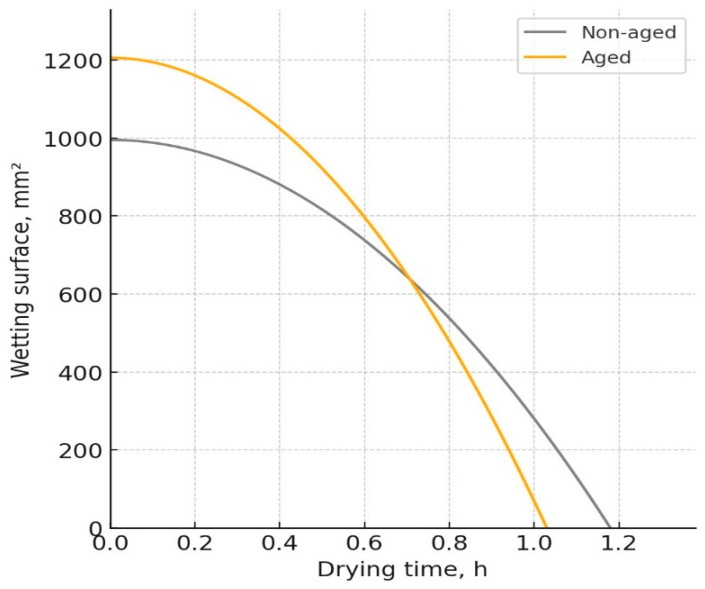
W-D profile of the fabric Ps1.

**Figure 5:**
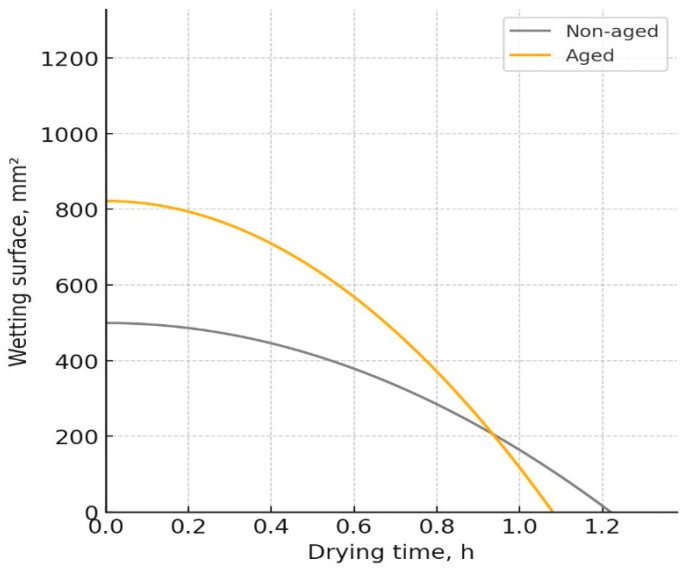
W-D profile of the fabric Ps2.

**Figure 6:**
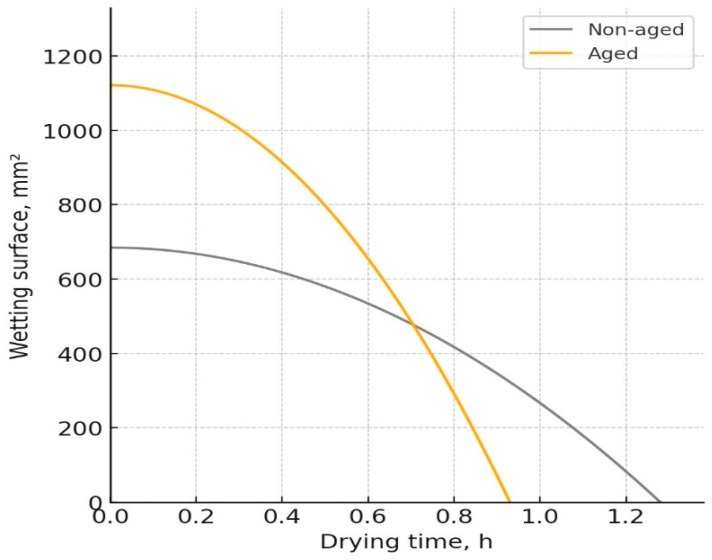
W-D profile of the fabric Ps3.

The W-D profiles of the analyzed fabrics, representing the relationship between the wetting surface of the fabric and its change over time due to the drying process, are presented in Figure 3, Figure 4, Figure 5 and Figure 6. The results indicated notable changes in the materials’ properties, reflecting the effects of aging on their performance. Before aging, material Pr (Figure 3) had a drying time of 0.86 h and a wetting surface of 956.5 mm^2^. After aging, the drying time decreased to 0.75 h, while the wetting surface increased to 1228.4 mm^2^. The increase in the wetting surface and the decrease in drying time after aging can be attributed to structural changes in the material. While aging leads to a reduction in porosity due to material shrinkage, the increase in fabric thickness contributes to a greater wetting surface, which is consistent with previous work [21]. The reduction in porosity leads to changes in moisture absorption and dispersion, affecting the drying time. Although the material becomes less porous, the increased fabric mass and thickness allow for faster moisture movement, reducing drying time.

For material Ps1, the drying time decreased from 1.18 h before aging to 1.03 h after aging, while the wetting surface increased from 995 mm^2^ to 1205.5 mm^2^ (Figure 4). Although the thickness of this material increased slightly after aging, the reduced drying time could be due to structural changes in materials (the shape of stitches), which allowed more efficient moisture evaporation from the surface.

Figure 5 illustrates that the drying time of material Ps2 decreased from 1.22 h before aging to 1.08 h after aging, while the wetted surface area expanded from 499.5 mm^2^ to 821.9 mm^2^. The substantial increase in the wetted surface suggests that aging induced structural modifications that enhanced moisture distribution. A slight increase in thickness implies that the aging process may have led to a more open fiber structure, promoting faster moisture evaporation and, consequently, a reduction in drying time.

Material Ps3 showed a reduction in drying time from 1.28 h before aging to 0.93 h after aging, with the wetting surface increasing from 684.4 mm^2^ to 1122.1 mm^2^ (Figure 6). The increase in thickness of this material indicated a possible swelling of the fibers and the accumulation of residues that may alter moisture absorption and drying properties.

The investigation revealed distinct changes in the wetting and drying behavior of the polyester materials after aging. All materials exhibited an increased wetting surface and reduced drying times due to aging. This suggests that the aging process induces structural modifications in the fabric structure, leading to changes in porosity and surface characteristics that facilitate faster moisture evaporation. In practical terms, such changes in the structure of football jerseys can influence critical performance aspects such as comfort, breathability, and moisture regulation. The increase in material thickness after aging can negatively affect the breathability, flexibility, and overall comfort of the clothing, which can reduce its functionality during intense physical activities. Therefore, understanding these changes is critical to developing sportswear that balances durability with optimal performance, ensuring that the material meets the physical demands of athletes while maintaining comfort.

### 3.4. Results of Bending Stiffness Testing

**Figure 7:**
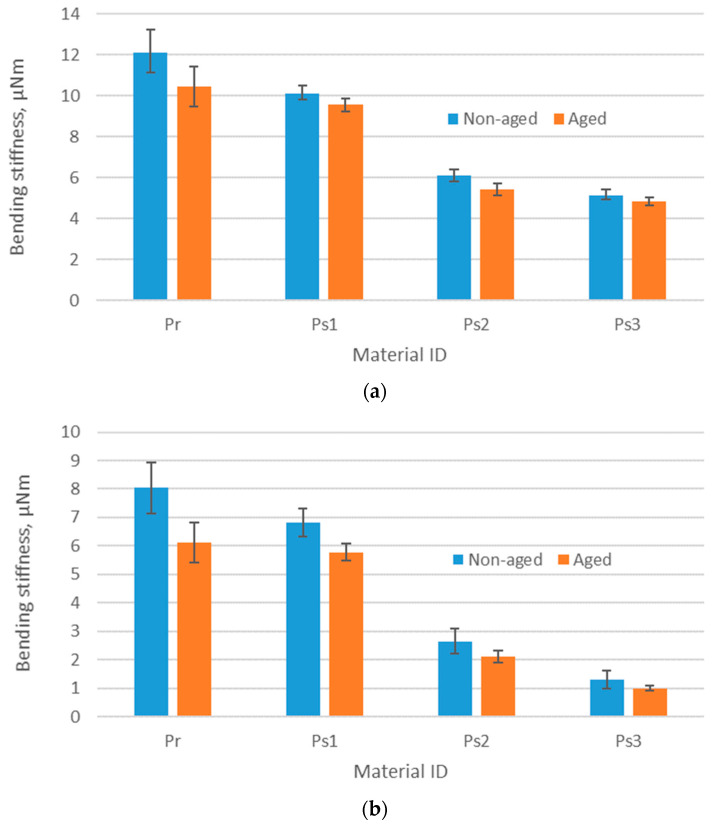
The bending stiffness of the fabrics tested in the direction of (**a**) wales and (**b**) courses.

Figure 7 presents the results of the material bending stiffness measured in two directions—the direction of wales (W) and courses (C). The measurement was carried out for both non-aged and aged materials. The bending stiffness of the tested materials ranges from 1.01 to 12.11 μNm. When comparing the influence of different yarn types (conventional vs. recycled), fabrics made from recycled polyester exhibit the highest bending stiffness in both non-aged and aged states, with values between 6.11 and 12.11 μNm, indicating a relatively stiff fabric. This finding aligns with previously published research results conducted by Telli and Ozdil [38], who demonstrated that a higher proportion of synthetic fibers in a blend with natural fibers leads to higher bending stiffness. However, their study did not specifically compare the stiffness of 100% conventional and recycled synthetic fibers. For fabrics made from conventional polyester yarn, the stiffness follows a mass-dependent hierarchy for non-aged materials. Specifically, the fabric labeled Ps1, which has the highest nominal mass per unit area among the three conventional polyester fabrics, also shows the highest bending stiffness. This observation is consistent with the findings of Yüksekkaya et al. [39], who reported that fabrics made from finer yarns, resulting in lower mass per unit area, tend to have lower bending stiffness. Additionally, there is a noticeable difference in bending stiffness depending on the stitch direction (wales vs. courses). In all cases, the stiffness is lower when tested in the course direction. This can be attributed to the anisotropic mechanical behavior of knitted structures. Namely, the stitch columns formed in the direction of wales provide structural reinforcement that helps to distribute and withstand the stress applied when the fabric is bent. Without these reinforcing columns, the fabric is more flexible and less resistant to bending in the course direction.

The overall effects of aging revealed a general decrease in the bending stiffness of all fabrics. A paired sample *t*-test (shown in Table 5) comparing the mean values of non-aged and aged samples demonstrated a very strong positive correlation between the two variables, with a Pearson correlation coefficient of 0.9989. Additionally, the *p*-value from the one-tailed test (*p* = 0.0321) indicated a statistically significant difference between the two means. The reduction in stiffness after aging can be attributed to repeated care processes, which cause fiber wear, making the fabric softer and less stiff. Moreover, frequent folding and bending during use lead to mechanical fatigue, further reducing stiffness over time. A previous study by Sachdeva et al. [40] found that artificial aging had varying effects, depending on the fiber composition of fabrics. Specifically, wool fabric showed minimal stiffness changes, cotton fabric experienced an 80% decrease, and silk fabric exhibited up to a 24% increase in stiffness. However, that study did not examine the impact of aging on synthetic fabrics, so the study presented in this paper complements the knowledge about this group of materials. In this study, the changes in polyester fabrics due to aging were less pronounced, with stiffness reductions ranging from 5% to 24%. The results indicated that the material made from recycled polyester yarn (denoted as Pr) demonstrated the highest stiffness among all materials tested, both before and after aging. This makes it particularly well-suited for applications that demand greater rigidity. In contrast, the material composed of conventional polyester yarn, with the lowest mass per unit area (referred to as Ps3), exhibits the lowest stiffness values. As a result, Ps3 is expected to offer greater flexibility, which may facilitate enhanced freedom of movement for athletes. This increased flexibility is also likely to improve wearing comfort and tactile perception—attributes that are increasingly acknowledged in research as critical to user satisfaction and athletic performance [41,42].

### 3.5. Results of Abrasion Testing

**Figure 8:**
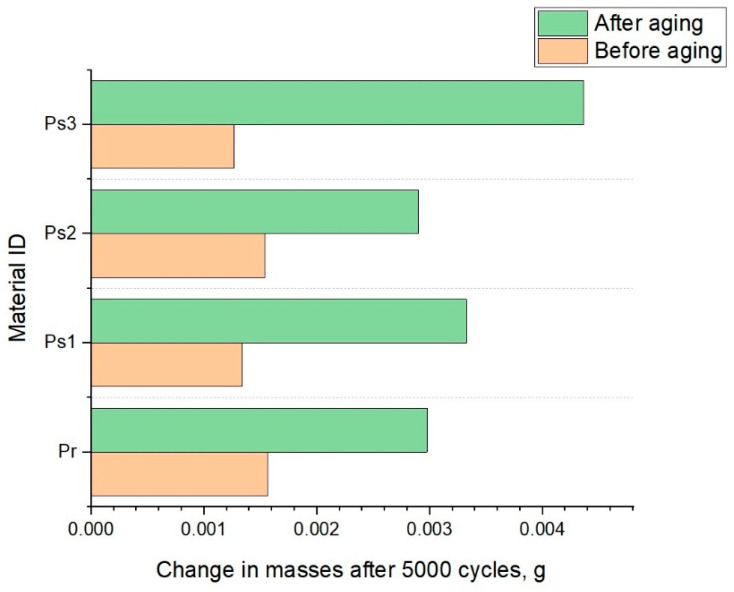
Results of the mass change after 5000 abrasion cycles.

Figure 8 presents the changes in the mass of the knitted fabric before and after 5000 abrasion cycles. The tests were performed before and after aging. To eliminate interference from external fibers originating from the wool-based abrasive material, all specimens were air-blown after abrasion to remove any loose material from the fabric surface.

The results show that abrasion caused a measurable decrease in surface mass across all samples, indicating fiber loss due to mechanical wear. Prior to aging, the highest mass reduction was recorded in the Pr sample before aging (Δ = 0.00156 g), suggesting lower structural resistance to surface degradation. Conversely, Ps3 exhibited the smallest change (Δ = 0.00127 g), indicating better initial cohesion and abrasion resistance.

After the aging process, a different trend was observed. The Ps3 sample exhibited the largest mass loss (Δ = 0.00436 g), indicating increased susceptibility to fiber removal after exposure to repeated washing and drying cycles. On the other hand, Ps2 showed the lowest mass reduction after aging (Δ = 0.00290 g), suggesting that its structural integrity remained more preserved after aging. These variations can be attributed to differences in the structural configuration of the knitted loops and the aging-induced relaxation of the fibers. As previously reported, aging resulted in an increase in mass, particularly in Ps1 and Ps2, potentially leading to changes in the surface fiber stability. The more pronounced loss of mass after aging in Ps3 might be a consequence of aging-induced fiber embrittlement or loosening of the fabric surface, despite its favorable initial behavior.

Given that these materials are intended for sportswear, where abrasion resistance plays a critical role in product durability, these findings emphasize the importance of evaluating both pre- and post-aging performance. Notably, the recycled polyester sample (Pr), while exhibiting greater mass loss before aging, demonstrated improved post-aging resistance relative to Ps3, indicating the potential for durability when subjected to long-term use.

The visual assessment of the surface condition of the knitted fabrics before aging and abrasion, after 5000 abrasion cycles (before aging) and after 5000 abrasion cycles (after aging), is shown in Table 6. Microscopic images were captured for each condition and sample, accompanied by pilling grades ranging from 1 to 5. These grades represent the extent of surface damage, with a grade of 5 indicating no visible change and a grade of 1 indicating severe pilling and extensive surface fuzzing. The average grades reflect increasing levels of fuzz and pill formation. Microscopic evaluation revealed notable changes in all specimens tested. After 5000 abrasion cycles, visible differences were observed regardless of the aging condition. In all specimens, fiber protrusion and fuzz accumulation were present, with significantly more pronounced effects in the after-aging condition. A common observation was the darkening of the fabric surface after aging, particularly following abrasion, likely due to structural changes caused by UV exposure and repeated washing. Despite air-blowing to remove loose fibers from the wool abrasive material, residual fibrils remained embedded in the knitted structure, more frequently and prominently in aged specimens. Among all tested materials, the recycled polyester (Pr) exhibited the most extensive surface changes. Even before aging, visible fuzz covered the surface. However, after aging, the fiber protrusion intensified and spread uniformly, leading to the lowest visual grade. This behavior may be related to the altered internal structure and reduced cohesion of recycled fibers, making them more prone to fiber fragmentation and mechanical damage. In contrast, specimen Ps2 showed the least visible surface changes, both before and after aging, maintaining a relatively smooth appearance. After aging, a slight darkening was observed, but pilling remained limited. This favorable outcome may result from its higher compactness, which provided greater resistance to mechanical disruption. Overall, the results highlight that aging contributes to greater visual degradation of the fabric’s surface, especially in terms of fiber loosening, discoloration, and pilling tendency.

These findings are of crucial importance for sportswear, as the aesthetic durability, the smoothness of the surface, and comfort have a direct influence on the performance of the clothing. The evaluation confirms that careful material selection and an assessment of durability under simulated real-life use conditions are essential to ensure the long-term functionality of athletic apparel.

### 3.6. Results of Elongation Properties

**Figure 9:**
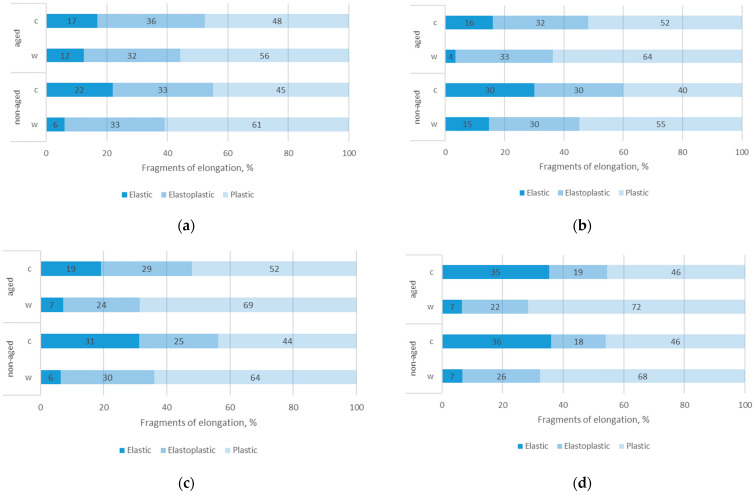
The fragments of the elongations of the tested fabrics in the direction of wales (w) and courses (c): (**a**) fabric Pr, (**b**) fabric Ps1, (**c**) fabric Ps2, (**d**) fabric Ps3.

Figure 9 shows the distribution of the elastic, elastoplastic, and plastic fragments of total elongation under different conditions (non-aged and aged) and test directions (wales and courses) for four different specimens. The data show that, for all non-aged specimens, the elastic region was more prominent in the course direction compared to the wales. This observation is consistent with a previously conducted study [43]. More specifically, the elastic region in the course direction was twice as large in specimen Ps1, 3.5 times as large in specimen Pr, and 5 times as large in specimens Ps2 and Ps3. The elastoplastic region remained relatively consistent (25–33%), except for specimen Ps3, where it was smaller in the course direction than in the wale direction (18% versus 26%). By exposing the sample to aging, the elastic region in the direction of the courses continued to be larger than in the direction of the waves, although this difference was less pronounced compared to non-aged samples. The elastoplastic property remained relatively stable with aging, which is consistent with previous work [44], where polyester material was also investigated. The most significant changes in the elastic region with aging were observed in sample Ps1, where the elastic region decreased notably by 73% in the wale direction and 46% in the course direction. Additionally, the plastic component generally increased with aging, suggesting that the materials became more plastic with time.

## 4. Conclusions

In this paper, the focus was placed on the changes in the physical–mechanical properties of materials used for football sportswear due to material aging. This study examines how aging affects important performance parameters by simulating real-life usage settings. The experimental results show that fabric aging leads to a reduction in porosity in both recycled and conventional polyester materials, which is due to the dimensional shrinkage of the material. Among the materials tested, recycled polyester had the highest porosity value. Aging also contributed to increased fabric thickness, reduced drying time, and increased wetting time, effects that are related to the reconfiguration of the microstructure. As far as the bending stiffness is concerned, the results show that recycled polyester had the highest stiffness, making it suitable for applications requiring stiffer materials. In contrast, the fabric made from conventional polyester with the lowest mass per unit area offered greater flexibility, which increased comfort during body movements. As the material ages, its abrasion resistance decreases. Remarkably, although the recycled polyester sample showed a greater loss of mass before aging, it showed improved abrasion resistance after aging, indicating potential durability for long-term use. Finally, the study demonstrated that, in most cases, plasticity increased as a result of aging, which indicated that the materials tend to become more plastic with aging.

The improvements in the development of high-quality sports materials are crucial, as these materials must withstand the stresses of use and provide the necessary comfort and protection for athletes. The results of this research contribute to the optimization of sportswear materials to ensure that the garments maintain their functional properties and durability throughout their useful life.

## Figures and Tables

**Table 1:** Overview of selected knitted materials and their basic structural properties.

Material ID	Mass per Unit Area, g/m^2^	Thickness, mm	Stitch Density—Horizontal/Vertical, stitches/cm	Structure
Recycled polyester—Pr	133	0.451	16/16	Single jersey
Standard polyester—Ps1	141	0.618	16/21	Double jersey mesh
Standard polyester—Ps2	132	0.491	19/18	Interlock
Standard polyester—Ps3	147	0.556	18/20	Double jersey mesh

**Table 2:** Environmental conditions according to the European Meteorological Center; weather forecast model HRES.

Average Value of the Environmental Parameter	Value
Air temperature	25 ± 2 °C
Relative humidity	66 ± 5%
Air pressure	1016 ± 2 hPa
Wind speed	4 ± 2 m/s
UV index	4 ± 2

**Table 3:** The mass per unit area of materials before and after aging.

Material ID	Mass per Unit Area Before Aging, g/m^2^	Mass per Unit Area After Aging, g/m^2^	Absolute Change, g/m^2^	Relative Change, %
Pr	133	136	3	2.26
Ps1	141	149	8	5.67
Ps2	132	143	11	8.33
Ps3	147	148	1	0.68

**Table 4:** The thickness of materials before and after aging.

Material ID	Thickness Before Aging, mm	Thickness After Aging, mm	Absolute Change, mm	Relative Change, %
Pr	0.451	0.481	0.030	6.65
Ps1	0.618	0.644	0.026	4.21
Ps2	0.491	0.512	0.021	4.28
Ps3	0.556	0.587	0.031	5.58

**Table 5:** The results of the *t*-test: paired two samples for means (bending stiffness of non-aged and aged materials).

*t*-Test: Paired Two Samples for Means	
Variance	7.6382
Pearson correlation	0.9989
Hypothesized mean difference	0
df	2
*t* stat	3.7575
P (*T* ≤ *t*) one tail	0.0321
*t* critical one tail	2.9199
P (*T* ≤ *t*) two tail	0.0641
*t* critical two tail	4.3027

**Table 6:** Microscopic images of the tested knitted fabrics before and after abrasion and aging with corresponding pilling grades.

Sample ID	Original Condition (Before Aging and Abrasion)	After 5000 Abrasion Cycles (Before Aging)	Pilling Grade	After 5000 Abrasion Cycles (After Aging)	Pilling Grade
Pr	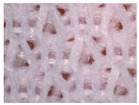	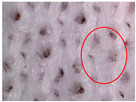	3	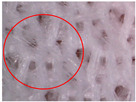	2
Ps1	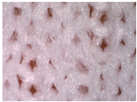	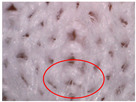	3	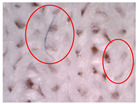	2
Ps2	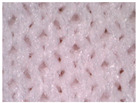	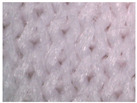	4	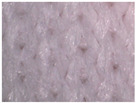	4
Ps3	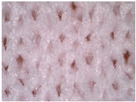	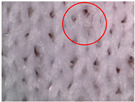	3	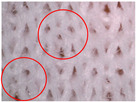	3

Legend: 5—no change, 4—slight surface fuzzing and/or partially formed pills, 3—moderate surface fuzzing and/or moderate pilling and pills of varying size and density partially covering the specimen surface, 2—distinct surface fuzzing and/or distinct pilling and pills of varying size and density covering a large proportion of the specimen surface, 1—dense surface fuzzing and/or severe pilling and pills of varying size and density covering the whole of the specimen surface.

## Data Availability

The data that support the findings of this study are available from the corresponding author upon reasonable request.
